# Key Management Scheme Based on Route Planning of Mobile Sink in Wireless Sensor Networks

**DOI:** 10.3390/s16020170

**Published:** 2016-01-29

**Authors:** Ying Zhang, Jixing Liang, Bingxin Zheng, Shengming Jiang, Wei Chen

**Affiliations:** 1 College of Information Engineering, Shanghai Maritime University, Shanghai 201306, China; yingzhang@shmtu.edu.cn (Y.Z.); liangjixing501@163.com (J.L.); zhengbingxin501@163.com (B.Z.); smjiang@shmtu.edu.cn (S.J.); 2 Department of Computer Science, Tennessee State University, Nashville, TN 37209, USA

**Keywords:** wireless sensor networks, key management, mobile sink, route planning, elliptic curve cryptography

## Abstract

In many wireless sensor network application scenarios the key management scheme with a Mobile Sink (MS) should be fully investigated. This paper proposes a key management scheme based on dynamic clustering and optimal-routing choice of MS. The concept of Traveling Salesman Problem with Neighbor areas (TSPN) in dynamic clustering for data exchange is proposed, and the selection probability is used in MS route planning. The proposed scheme extends static key management to dynamic key management by considering the dynamic clustering and mobility of MSs, which can effectively balance the total energy consumption during the activities. Considering the different resources available to the member nodes and sink node, the session key between cluster head and MS is established by modified an ECC encryption with Diffie-Hellman key exchange (ECDH) algorithm and the session key between member node and cluster head is built with a binary symmetric polynomial. By analyzing the security of data storage, data transfer and the mechanism of dynamic key management, the proposed scheme has more advantages to help improve the resilience of the key management system of the network on the premise of satisfying higher connectivity and storage efficiency.

## 1. Introduction

In traditional static wireless sensor networks (WSNs), all static nodes transmit data to a sink node or base station in a multi-hop manner. Therefore, the nodes which are close to the base station or sink node usually need to take on a tremendous data forwarding task. This will lead to nodes’ premature death due to the excessive energy consumption that easily forms “energy holes”, and eventually affects the normal operation of the entire network. Thus, how to prolong the survival time of the network becomes the key problem to be studied in resource-constrained WSNs. Studies show that dynamic clustering and the introduction of Mobile Sinks (MSs) can better achieve the result of balancing the nodes’ energy consumption [1,2]. The communication patterns in WSNs can be divided into single hop transmission, multi-hop transmission and assistant cache transmission [3,4]. In the assistant cache transmission model, the sensed data can be transmitted to auxiliary nodes in single hops or multi-hops, and the MS only needs to traverse the auxiliary nodes within a single hop instead of communicating with all nodes in the network. The model reduces multi-hop data transmission and avoids causing energy holes as much as possible. In order to better improve some aspects of the performance of WSNs, they need to take full advantage of the MS mobility. Therefore, it is important to choose the mode and plan the mobile route for MS’s movement. There are several mobile modes for a sink node, including random movement, desired trajectory movement, controlled movement and choosing the mobile location by using an optimization algorithm, *etc.* The introduction of the the dynamic clustering concept and MS movement planning will have a great influence on key management of wireless sensor networks, and it extends static key exchange to dynamic key exchange.

The proposed scheme determines the MS’s traversal path by using a Genetic Algorithm (GA), and fully considers the relative shortest path and the probability of path selection based on the topological structure of dynamic clustering in the wireless sensor network. In the scenario with a MS, the cluster head and MS establish a session key by the improved Diffie-Hellman (ECDH) key agreement. Meanwhile, the member nodes with limited resources set up a session key with the cluster head by using the binary symmetric function. Compared with some existing key management schemes, the proposed scheme has better connectivity, storage effectiveness, safety and energy consumption advantages.

The rest of this article is organized as follows: in Section 2, some background and related work on MS and key management in sensor networks are introduced. Then, in Section 3, we describe the design of the proposed scheme in detail. Section 4 presents the simulation and results analysis, and finally we conclude the paper in Section 5.

## 2. Related Work

MSs can prolong the survival time of WSNs and balance the nodes’ energy consumption. For MS route planning, some research conclusions had been reached by other scholars. Xing *et al.* [5] proposed a network model in which nodes deliver data to the nearest fixed sink node with multiple hops, and then the MS collects data by traversing all the fixed sink nodes. This scheme turns the problem of MS route planning into a Traveling Salesman Problem (TSP) with the limitations of the MS’s moving speed and node’s memory space. However, this method needs to visit all the sink nodes, so it will increase the MS movement path, and fails to make full use of the communication capacities among the nodes. By gridding the network, Gao *et al.* [6] divided the network into several virtual network points, and elect the cluster head based on the weighted sum of the residual energy and the distance from the nodes to the center of gravity of the cluster. This can avoid the nodes with lower energies from becoming the cluster head. Finally the sink nodes can be scheduled to receive the collected data from the cluster heads by the controllable moving strategy, and this can save the energy consumption of the network. Thanigaivelu *et al.* [7] used the grid clustering method, in which the main cluster head and the deputy cluster head are introduced to communicate and collect data, and the sink node collects data along with the scheduled centers of the grids. Kumar *et al.* [8] limited the distance between each node and the cluster center within the maximum communication distance, and then the detection area will be clustered. The MS visits the cluster center for data collection according to the optimal moving path using a TSP method. Rao *et al.* [9] considered the influences of energy consumption and data delay on the process of route choice, and a mathematical model was built for the relationship of energy consumption and data delay based on boundary hop counts.

Guo *et al.* [10] proposed the disk clustering method. The data collection points were selected according to nodes’ specific locations, and the MS will visit the collection points by the optimal route which is solved by a quantum genetic algorithm. All the above research only considers the routing protocol and data collection based on the optimal MS path planning, rather than comprehensively considering the network energy consumption, the survival time and any safety issues, *etc.*

The core of the security problem of WSNs is how to safely establish the session keys, which is the vital process to ensure the safety of data collection and transmission. For the sensor nodes whose resources are constrained, the omplex encryption algorithms used in traditional networks cannot be directly transplanted to a WSN. Therefore, under the premise of network security, lightweight schemes should be considered preferentially. Symmetric key systems and asymmetric key systems are widely used in technology encryption systems. Random key pre-distribution and pre-allocated polynomial keys are typical methods used in symmetric key systems. In the Eschenauer and Gligor (EG) scheme [11], partial keys are randomly selected from a key pool generated by the server to build the key ring. The nodes can set up a secure communication link when the adjacent node finds that the same key exists in the key ring. Liu *et al.* [12] proposed an improved random key distribution scheme called *q*-composite key pre-stored scheme. This scheme requires that security key links can be set up only when adjacent nodes share at least *q* identical keys. Obviously, this scheme improves the resilience ability of the network, but the redundancy of keys increases, and it is not conductive to the expansion of the network. Amar *et al.* [13] proposed the Authentication and Pairwise (AP) key scheme. The scheme improves the effectiveness and safety of nodes’ storage by making use of the resource advantages of high-end nodes in heterogeneous sensor networks and adopting the method of asymmetric key pre-distribution. Hussain *et al.* [14] presented a polynomial key pre-distribution scheme. The key pairs can be set up by *t*-degree binary symmetric polynomials generated by the server. The network will not disclose any information when the number of captured nodes is less than *t*. Huang *et al.* [15] proposed a key pre-distribution scheme based on a polynomial pool on the basis of combining the concepts of key pool and a polynomial pre-distribution scheme. The scheme increases the network security, but the requirements of storage space and computing ability are higher when the degree of the polynomial *t* becomes larger. The key management scheme based on elliptic curves is a typical asymmetric key system method. Villas *et al.* [16] and El-Moukaddem *et al.* [17] introduced the elliptic curve concept into public key systems, and the security could be translated into the problem of a discrete logarithm based on elliptic curves. It has been proved that Elliptic Curve Cryptography (ECC) can be applied in key management of WSNs effectively [18]. Its key length is shorter than that of other public key systems while achieving the same safety indexes, and its amount of calculation and the required storage space are all smaller.

## 3. The Scheme Design

### 3.1. Clustering Deployment

In order to address the problem of energy holes, ensuring a balanced energy consumption, while improving the network coverage rate, on the basis of the method by Latiff *et al.* [19], we added a new optimal object: coverage factor to the fitness function, which can improve the network coverage on the premise of energy equilibrium. The clustering is performed by a base station, and the fitness function is defined as follows:

(1) $$F=a_{1}\times f_{1}+a_{2}\times f_{2}+(1-a_{1}-a_{2})\times f_{3}$$

(2) $$f_{1}=\underset{k=1,2\cdots K}{\max}\left\{\sum_{\forall n_{i}\in C_{k}}d(n_{i},C_{k})/Num_{C_{k}}\right\}$$

(3) $$f_{2}=\sum_{i=1}^{N}E(n_{i})/\sum_{k=1}^{K}E(C_{k})$$

(4) $$f_{3}=\sum_{k=1}^{K}N_{k}$$

where *f*_1_ is the maximum average Euclidean distance between nodes and their associated cluster head, *d*(*n_i_*,*C_k_*) is the distance between node *n_i_* and its cluster head *C_k_*, *Num_Ck_* is the number of nodes which belong to cluster *C_k_*, *f*_2_ is the ratio of total initial energy of all the nodes *n_i_*(1,2,3,…,N) in the network to the total current energy of all cluster heads in the current round, *f*_3_ is the number of the nodes whose distances to their associated cluster head are larger than the maximum communication range. *a*_1_, *a*_2_ are user-defined evaluation weight coefficients used to weigh the contribution of each sub-objective to the fitness function. By constructing the fitness function, we can effectively minimize the sum of the distances between the member nodes and their cluster heads, and also optimize the energy efficiency of the network.

In this clustering algorithm, the *K* candidate cluster heads could be abstracted into a particle, which constantly updates its position and velocity information and calculate their fitness values at different positions. That is to say, the operation is iterative and updates the positions of cluster heads, and finds the optimal position of cluster heads which can balance the energy consumption and improve the coverage rate of the network. By this method, we can convert the sensor network clustering problem into a mathematical iterative optimization problem. The specific process of the clustering algorithm can be summed up as follows:

1. Initialize velocity and position of each particle *P_i_* (*i* = 1, 2, 3,…, S), and each particle contains *K* candidate cluster heads.
2. Assign node *n_i_* to the nearest cluster head, and then calculate the fitness value of each particle *P_i_* (*i* = 1, 2, 3, …, S) by Equations (1)–(4).
3. Ensure the individual optimal solution *P_i_* (*t*) and the global optimal solution *G* (*t*) for each particle.
4. Update the particle’s velocity and position.
5. Map the particle’s location to the position of cluster heads.
6. Repeat the above steps 1–5 until the maximum number of iterations is reached or the certain required coverage rate is satisfied.

After clustering, the network gets into steady stage. Sensor nodes transmit the collected data to their cluster heads with single hop. The pseudo code of the clustering algorithm is as follows:

  Random initialization for velocity $v_{i}(0)$ and position $x_{i}(0)$ of particles i∈[1,2,⋅⋅⋅,S]  Map the particle location to position of the nodes according to the distances between the positions of each particle and the nodes  **for** each particle i∈[1,2,⋅⋅⋅,S]:  Calculate fitness $f_{i}(0)$ of particle by Equations (1)–(4)    $P_{i}(0)\leftarrow f_{i}(0)$**end for**$G(0)\leftarrow \min\{f_{1}(0),f_{2}(0),\cdots ,f_{S}(0)\}$**for** iterations t∈[1,2,⋅⋅⋅,MaxIter]:  **for** each particle i∈[1,2,⋅⋅⋅,S]:    Update $v_{i}(t)$ and $x_{i}(t)$    Map the particle location to the position of the eligible cluster head candidates    Calculate the fitness of particles    **if**
$f_{i}(t)<P_{i}(t)$      **then**
$P_{i}(t)\leftarrow f_{i}(t)$    **if**
$P_{i}(t)<G(t)$      **then**
$G(t)\leftarrow P_{i}(t)$   **end for****end for**

### 3.2. Mobile Route Planning

**Figure 1:**
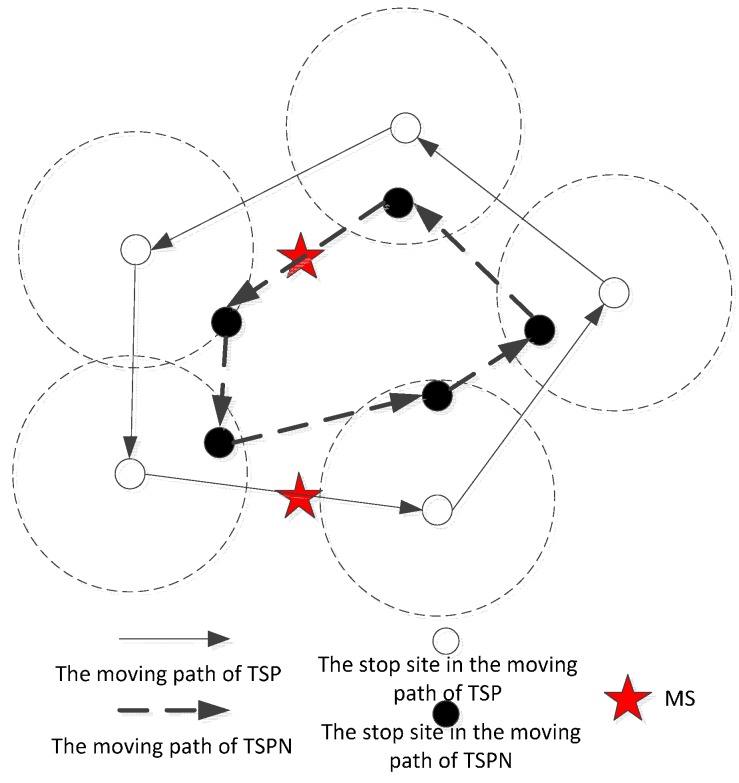
The schematic of mobile route for MS in TSPN.

In traditional wireless sensor networks, cluster heads directly disseminate data to the base stations after completing data collection and fusion. The greatest disadvantage of directly transmitting the data to the base station from the cluster heads is that sometimes thius causes a larger energy consumption due to the long transmission distances. In this article, MS visits all cluster heads, which can shorten the data transmission distance of cluster heads, and meanwhile it can guarantee the quality of communication links, so the MS path planning problem becomes how to choose the optimal traversal path. Since a sensor node itself has a certain ability of communication, the MS does not have to move to the exact location of cluster heads for data collecting. It only needs to enter the communication range of the cluster heads, and then it implements the data interaction with the cluster heads. Therefore, this kind of mobile model can be abstracted as a TSP problem with neighbor areas, hence we name this kind of model the Traveling Salesman Problem with Neighbor areas (TSPN). A schematic of this kind of mobile route for MSs is shown in Figure 1.

This scheme addresses the problem of path planning in TSPN by using a Genetic Algorithm due to its good global optimization search capability. The optimization goal is to make the MS first visit the cluster head whose cluster scale is larger, and simultaneously try to reduce the distance a MS has to move.

**Figure 2:**
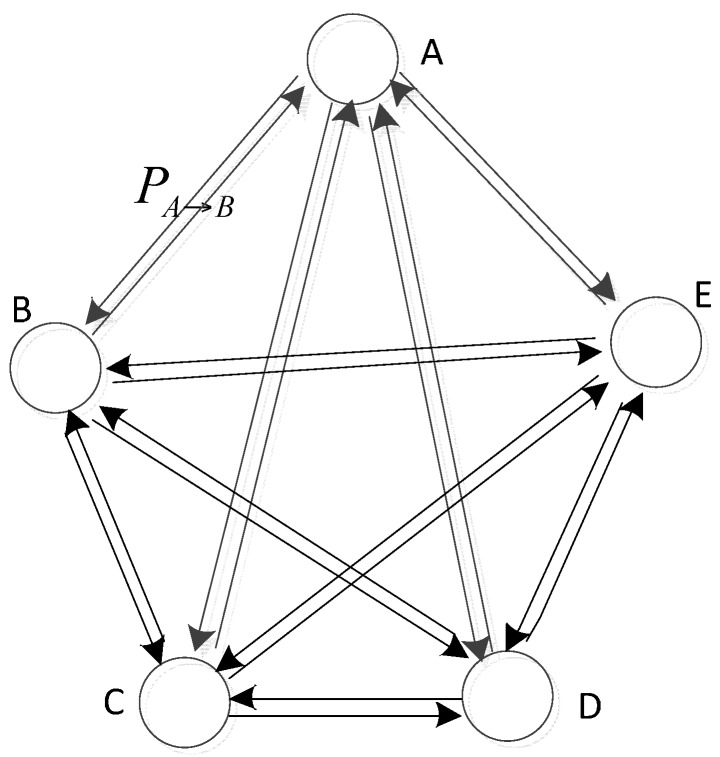
Probability of route selection for MS in TSPN.

As shown in Figure 2, the probability *P_A→B_* which presents the probability that a MS moves from cluster head *A* to the next stop cluster head *B* is defined as Equation (5):

(5) $$P_{A\to B}=\frac{Num_{C_{B}}}{\sum_{k\neq A}^{K}Num_{C_{k}}}$$

where *Num_C_B__* is the number of nodes in cluster *B*, and $\sum_{k\neq A}^{K}Num_{C_{k}}$ is the number of all nodes except for the nodes in cluster *A*. The fitness function is defined as follows:

(6) $$F={\left(b\times f_{1}+(1-b)\times f_{2}\right)}^{m}$$

(7) $$f_{1}=1-\frac{len(i)-minlen}{maxlen-minlen}$$

(8) $$f_{2}=\frac{pro(i)-minpro}{maxpro-minpro}$$

where *f*_1_ is the distance factor which impacts the movement of MS, *len*(*i*) (*i* = 1, 2, …, *n*) is the sum of distances for all the populations, *maxlen* is the maximum sum of route distances among all the populations, *minlen* is the minimum sum of route distances among all the populations, *f*_2_ is the probability of the route choice factor which influences the movement of *MS*, *pro*(*i*) (*i* = 1, 2, …, *n*) is the sum of probabilities of the route choice for all the populations, *maxpro* is the maximum sum of the probabilities of the route choice among all the populations, *minpro* is the minimum sum of the probabilities of the route choice among all the populations, *b* is the weight coefficient, *m* is acceleration index to eliminate the fitness value, and it should not be given a larger value.

The specific process of the route selection algorithm can be summed up as follows:

1. Generate initial populations. The traversal sequence of cluster heads by MS is regarded as the coding of the algorithm. The initial route combination is a 1 × *N* scale matrix (*N* contains (*N* – 1) cluster heads and a base station) generated randomly, and *n* initial populations are generated.
  Build distance matrix *D* and probability matrix *P*. *D*(*i,j*) denotes the distance between cluster head *i* and cluster head *j*, and $D(i,j)=\sqrt{{\left(x_{i}-x_{j}\right)}^{2}+{\left(y_{i}-y_{j}\right)}^{2}}$. *P*(*i,j*) is the probability of route choice, and $P(i,j)=Num_{C_{j}}/\sum_{k\neq i}^{K}C_{k}$.
2. Calculate the total length of all individuals path: *len*(*i*) and the total probability of route selection: *pro*(*i*).
3. Calculate the fitness values of all individuals by using Equations (6)–(8). The smaller the sum of distances is, the greater the total probability of route selection and the fitness value will be.
4. Selection operation. The individual will be selected if the fitness *F* is great than random value *r*.
5. Cross operation. Partial match cross is adopted if the cross probability *p_c_* is great than a random value *r*.
6. Mutation operation. Two docking points in the route are randomly selected to exchange if the probability of mutation *p_m_* is great than random value *r*.
7. Update the populations.
8. Repeat steps 3–7 until the maximum number of iterations is reached

**Figure 3:**
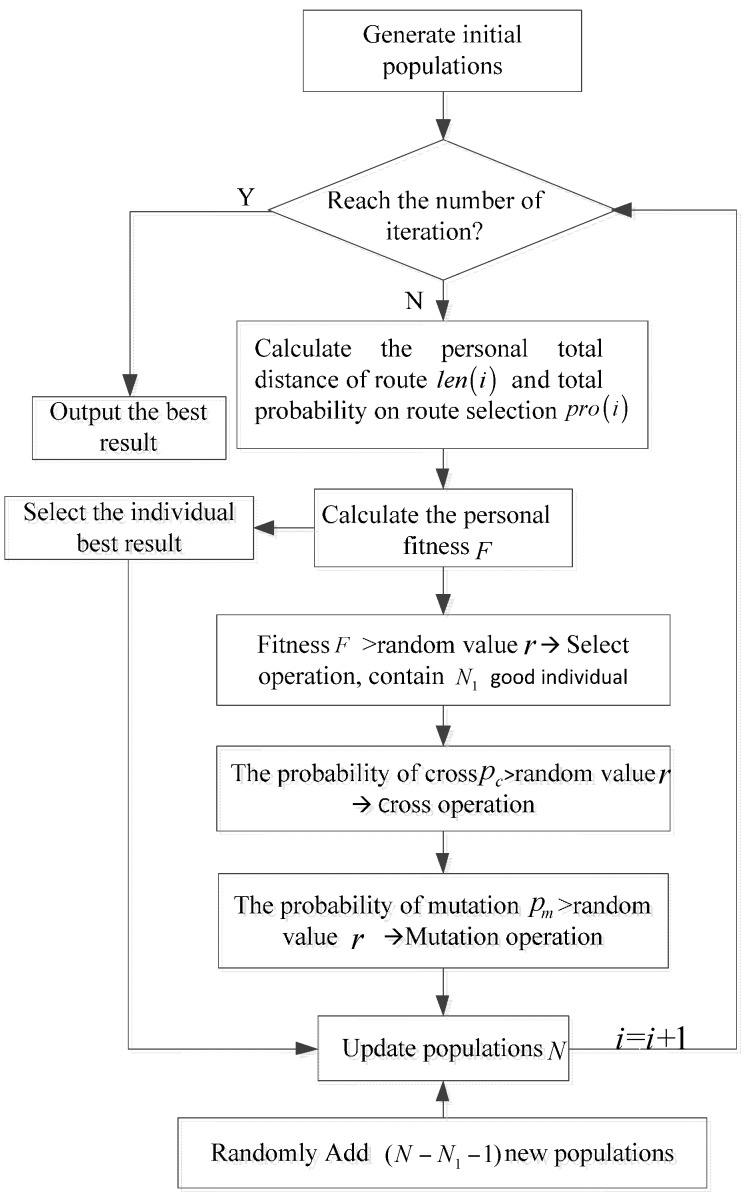
The flowchart of the route planning algorithm for the movement of MSs.

The flowchart of the route planning algorithm for the MS movement is shown in Figure 3.

### 3.3. Establishment of Session Keys

After the MS movement route is determined, the MS can interact with cluster heads for data transmission. Data interaction is under the premise of building secure session keys. In this article, since the MS contains relatively more abundant resources, the cluster head and MS can establish a session key with the improved ECDH algorithm. However, the member nodes and cluster head could establish a session key using a binary symmetric function due to the limited energies of the member nodes.

#### 3.3.1. Initial Stage

Before the node deployment, the server generates a pair of public key *P* and private key *S* based on ECC for each node, which satisfies the relationship: *P = S × G*, where *G* denotes a base point on the elliptic curve. Each member node has a pre-stored unique identifier *ID_Ni_*, a pair of public and private keys (*S_Ni_*,*P_Ni_*), a digital signature (*W_Ni_*,*w_Ni_*), the binary symmetric function *F*, and the hash function *H*. MS has a pre-loaded unique identifier *ID_MS_*, a pair of public and private keys (*S_MS_*,*P_MS_*). a digital signature (*W_MS_*,*w_MS_*), and the hash function *H*. The digital signature (*W_i_*,*w_i_*) [20] can be expressed as follows:

(9) $$\left\{ \begin{array}{l} W_{i}=r_{i}\times G=(x_{w_{i}},y_{w_{i}})\\ w_{i}={r_{i}}^{-1}(H(ID_{i}||T_{i})+S\times x_{w_{i}})(mod\;n) \end{array}\right.$$

where *r_i_* is a random value, *G* is a base point on the elliptic curve, *H* is the hash function, and *S* is the private key.

When node *i* sends a request for digital signature verification within time *T*, the legality of node *i* can be validated only if we can validate: *N_i_* = *W_i_*. The process of the validation can be expressed as follows:

(10) $$\begin{array}{ll} N_{i} & =w_{i}^{-1}H(ID_{i}||T)G+w_{i}^{-1}x_{w_{i}}P\\ & =w_{i}^{-1}(H(ID_{i}||T)+x_{w_{i}}S)G\\ & =w_{i}^{-1}r_{i}r_{i}^{-1}(H(ID_{i}||T)+x_{w_{i}}S)G\\ & =w_{i}^{-1}r_{i}\{r_{i}^{-1}(H(ID_{i}||T)+x_{w_{i}}S)\}G\\ & =w_{i}^{-1}r_{i}w_{i}G\\ & =W_{i} \end{array}$$

#### 3.3.2. Key Establishment Stage

##### The Establishment of Cluster Keys

The MS launches the request to establish a session key, and then cluster head *C_i_* will reply to the MS with the message: *C_i_*→*MS*:{*ID_Ci_*‖(*W_Ci_*,*w_Ci_*)‖*P_Ci_*} within a single hop. MS validates the legitimacy of cluster head *C_i_* by (*W_Ci_*,*w_Ci_*), and randomly generates a large integer *k* and a pair of elliptic curve scalars (*M*_1_,*M*_2_), where *M*_1_ = *k* × *G*, *M*_2_ = *K_cluster_* + *k* × *P_Ci_*, where *K_cluster_* = *H*(*r*‖*ID_MS_*). MS unicasts the scalars to cluster head *C_i_*, and calculates the cluster key after verifying the validity of the MS:

(11) $$\begin{array}{ll} K_{C_{i}} & =M_{2}-S_{C_{i}}\times M_{1}\\ & =K_{cluster}+k\times P_{C_{i}}-S_{C_{i}}\times k\times G\\ & =K_{cluster} \end{array}$$

After the cluster key is encrypted with the public keys of the nodes in the cluster by the cluster head, the cluster head will broadcast the encrypted information within the cluster: *C_i_*→*:{*ID_Ci_*‖(*W_Ci_*,*w_Ci_*)‖*E*(*P_Ni_*,*K_cluster_*)}. The nodes in the cluster decode the cluster key *K_cluster_* with the corresponding private key after receiving the encrypted information and verifying the legality of the cluster head.

##### Session Keys Establishment Between the Cluster Heads and a MS

The agreement method of a session key between cluster head and MS is based on the improved ECDH algorithm, which introduces a session key factor on the basis of the ECDH algorithm [21,22], and the reciprocal of the private key will participate in the key agreement process. The specific steps are as follows:

- The cluster head randomly selects a large integer *R_Ci_* ∈ [1, *n* − 1], then calculates a session key factor *M_Ci_* = *R_Ci_* × *P_MS_* by utilizing the public key *P_MS_* which is opened by the MS, and sends it to the MS.
- The MS randomly selects a integer *R_MS_* ∈ [1, *n* – 1] after validating the legality of cluster head *C_i_*, then calculates session key factor *M_MS_* = *R_MS_* × *P_Ci_* by utilizing the public key *P_Ci_* which is uploaded by the cluster head, and transmits it to the cluster head *C_i_*.
- After receiving the session key factor from the MS, the cluster head calculates the session key *K_Ci–MS_* with the MS:
  (12) $$\begin{array}{ll} K_{C_{i}-MS} & =R_{C_{i}}\times M_{MS}\times S_{C_{i}}^{-1}\\ & =R_{C_{i}}\times R_{MS}\times P_{C_{i}}\times S_{C_{i}}^{-1}\\ & =R_{C_{i}}\times R_{MS}\times S_{C_{i}}\times G\times S_{C_{i}}^{-1}\\ & =R_{C_{i}}\times R_{MS}\times G \end{array}$$
- In a similar way, the MS calculates the session key *K_MSi–Ci_* with the cluster head after receiving the session key factor from the cluster head.
  (13) $$\begin{array}{ll} K_{MS-C_{i}} & =R_{MS}\times M_{C_{i}}\times S_{MS}^{-1}\\ & =R_{MS}\times R_{C_{i}}\times P_{MS}\times S_{MS}^{-1}\\ & =R_{MS}\times R_{C_{i}}\times S_{MS}\times G\times S_{MS}^{-1}\\ & =R_{MS}\times R_{C_{i}}\times G \end{array}$$

Due to the fact *K_Ci–MS_* = *K_MSi–Ci_*, the session key establishment between the cluster head *C_i_* and the MS is completed.

##### Session Keys Establishment Between a Cluster Head and the Member Nodes

Due to the limitations of member nodes’ resources, the cluster head establishing a session key with member nodes has to adopt the binary symmetric function, and the specific process is as follows:

- The cluster head sends the request to establish a session key with the member node and disseminates the digital signature (*W_Ci_*,*w_Ci_*). The member node replies with the digital signature (*W_Ni_*,*w_Ni_*), after validating the legality of the cluster head. In a similar way, after validating the legality of member nodes, the cluster head establishes a session key with the member nodes:
  (14) $$K_{C_{i}-N_{i}}=F(ID_{C_{i}}⊕K_{cluster},ID_{N_{i}}⊕K_{cluster})$$
- Member nodes validate the legality of the cluster head, and then set up a session key with the cluster head:
  (15) $$K_{N_{i}-C_{i}}=F(ID_{N_{i}}⊕K_{cluster},ID_{C_{i}}⊕K_{cluster})$$
  Due to the symmetry of binary function, we can get the expression: $K_{C_{i}-N_{i}}=K_{N_{i}-C_{i}}$
- A session key between a base station and a member node is also established by the binary symmetric function.

##### Traversal to Next Cluster Head for Session Key Establishment

A session key will be established in accordance with the above method when the MS visits the next cluster head. After the MS collects all the data from cluster heads and comes back to the base station, we have a complete data collection cycle.

#### 3.3.3. Key Revocation and Update

When the node's residual energy is less than a certain threshold, the base station will exclude the node from the network during the clustering process. Therefore, it is impossible for it to participate in the remainder of the process, including cluster head selection, path planning and session key establishment. If a normal node was captured, the intrusion detection mechanism will be used to find this situation, and the captured node will be noticed by the base station, and it will be prohibited from participating in establishing a session key in the next cycle. In this paper, the dynamic clustering scheme is adopted, which means the base station will launch re-clustering in every period of *T*. Due to the reselection of cluster heads, cluster keys and all kinds of session keys among the MS, cluster heads, member nodes, and base stations all need to be rebuilt, and all the key updates will be completed in this process. This ensures one session key will be used once time. It ensures the network uses a session key with a short time for communication, and it can reduce the risk of cracking for the network.

#### 3.3.4. New Nodes to Join In

The new node is preloaded with things like a unique identifier *ID_Ni_*, a pair of public and private key (*S_Ni_*,*P_Ni_*), a digital signature (*W_Ni_*,*w_Ni_*), the hash function *H*, and the binary symmetric function *F*. A new node sends its joining request to a base station, and after the base station receives the request, it will validate its legality by the signature (*W_Ni_*,*w_Ni_*). In the new round of clustering, the new node will be added to the associated cluster and establish the session keys with the cluster head or base station.

## 4. Simulation and Evaluation

In the simulation experiment, 100 nodes are randomly deployed in a 100 × 100 m^2^ area, and the base station is located in the center of the monitoring area. The server mentioned in the initial stage of key establishment generates a pair of public key *P* and private key *S* and will be appointed as the base station. In fact, the common sensor nodes in the network do not have enough computation and storage capacity, and the mobile sink node mainly takes on the tasks of sending, receiving and storage of data, and also the communication management with the cluster heads. Most of the computation in the network is undertaken by the server. In this implementation circumstance, the server is mainly implemented in the base station. Heinzelman *et al.* [23] proposed the scheme of using the wireless communication model. If we transmit *k* bits message and the distance is *d*, the node’s energy consumption can be calculated as follows:

(16) $$\begin{array}{ll} E_{Tx}(k,d) & =E_{Tx\_elec}(k)+E_{Tx\_amp}(k,d)\\ & =\left\{ \begin{array}{ll} kE_{elec}+k\varepsilon_{fs}d^{2} & d<d_{0}\\ kE_{elec}+k\varepsilon_{mp}d^{4} & d\geq d_{0} \end{array}\right. \end{array}$$

where, $\varepsilon_{fs}$ and $\varepsilon_{mp}$ are energy consumption coefficients of power amplification circuit, and $d_{0}=\sqrt{\varepsilon_{fs}/\varepsilon_{mp}}$.

When receiving *k* bits data, the energy consumption of data transmission can be calculated as follows:

(17) $$E_{Rx}(k)=E_{Rx\_elec}(k)=kE_{elec}$$

where *E_elec_* = 50 nJ/bit, $\varepsilon_{fs}$ = 10 pJ/bit/m^2^, and $\varepsilon_{mp}$ = 0.0013 pJ/bit/m^4^. The energy consumption of the data aggregation is set as: *E_DA_* = 5 nJ/bit. The parameters in the experiment are set up as in Table 1.

**Table 1:** Simulation Parameter Settings.

PSO Parameters	Parameter Values	GA Parameters	Parameter Values
The number of particles S	20	The number of populations n	100
Learning factors $c_{1},c_{2}$	2	Cross probability $P_{c}$	0.8
Inertia weight *w*	Decrease from 0.9 to 0.4 linearly	Mutation probability $P_{m}$	0.1
Evaluation factor $a_{1}$	0.3	Evaluation factor a	0.4
Evaluation factor $a_{2}$	0.4	Stops N	6
The number of cluster head K	5	Cross width W	3
The size of the message data	4000 bit	Acceleration index to eliminate fitness m	2
Initial energy of node $E_{0}$	0.1 J	The size of the message data	4000 bit
Communication radius $d_{\max}$	30 m	Initial energy of node $E_{0}$	0.1 J
		Communication radius $d_{\max}$	30 m

### 4.1. Network Connectivity

Considering the higher resource demand for nodes to use the asymmetric encryption algorithm, in this scheme, the cluster head establishes a session key with the MS by utilizing the improved ECDH algorithm, and the cluster head builds a session key with the member nodes based on the binary symmetric function. Since the MS traverses all the cluster heads within a period *T*, the key connectivity should be considered within the period time of *T*. According to the steps of all kinds of the session key establishment described above, member nodes in the cluster can set up a unique session key with a cluster head as long as it is within the communication range of the cluster head, so the key connectivity in the scheme depends on the network coverage rate after clustering. As shown in Figure 6, network connectivity can reach 100% when all the nodes are located within the range of a single hop to visit their associated cluster heads as long as the appropriate clustering method is adopted.

**Figure 4:**
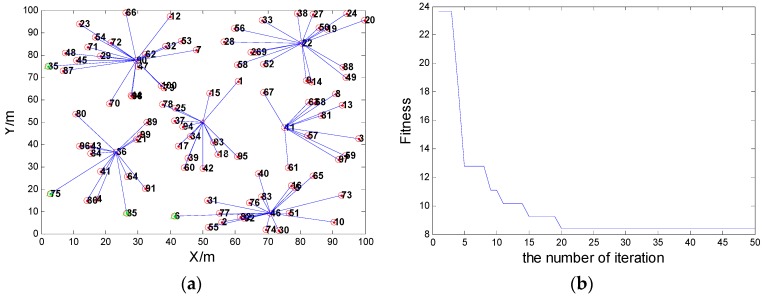
(**a**) The network connectivity when the communication radius R = 25; (**b**) The fitness value when the node communication radius R = 25.

**Figure 5:**
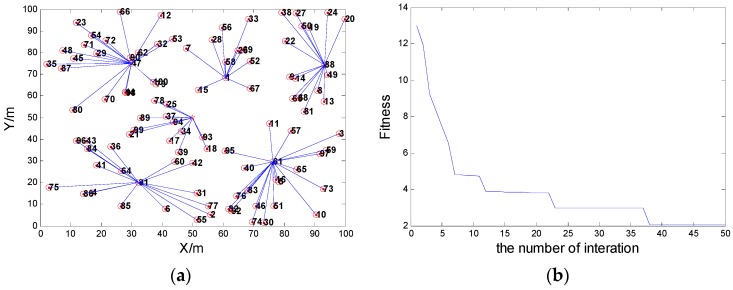
(**a**) The network connectivity when the communication radius R = 30; (**b**) The fitness value when the node communication radius R = 30.

1. When the communication radius R = 25, the coverage rate is 96% after clustering, and the network connectivity can reach 96%. In this scenario, with the proposed scheme, the network connectivity is shown in Figure 4a, and the variation of the fitness value is shown in Figure 4b.
2. When communication radius is R = 30, the coverage rate is 100%, and the network connectivity can reach 100%. In this scenario, with the proposed scheme, the network connectivity is shown in Figure 5a, and the variation of the fitness value is shown Figure 5b.

**Figure 6:**
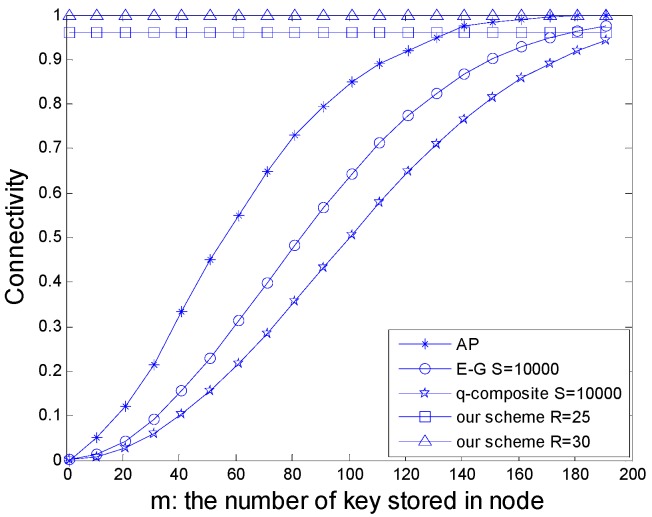
The comparison of network connectivity among the schemes of AP, E-G (s = 10,000), *q*-composite (s = 10,000), and the proposed scheme (R = 25 and 30).

The comparison of the schemes, AP, E-G (s = 10,000), *q*-composite (s = 10,000), and the proposed scheme (R = 25 and 30), is shown in Figure 6.

We can conclude from above, with the same number of keys stored in the nodes, the proposed scheme has higher keys connectivity efficiency compared with the E-G scheme, *q*-composite scheme, and the AP scheme.

### 4.2. Keys Storage Overhead

After completing the process of establishing a session key, a cluster head needs to store some keys: cluster key, a pair of public and private keys of the cluster head, the public keys of all nodes in the cluster, the session keys with all nodes in the cluster, and the session key with the MS. A member node also needs to store some keys: cluster key, a pair of public and private keys of itself, and the session key with the associated cluster head. Therefore, the total storage overhead is 4(M+1)+2N+4N, where *M* denotes the number of cluster heads, *N* is the number of member nodes. In the AP scheme, the number of member nodes is 1000, every node is preloaded with 20 keys, and the number of senior nodes is 20, and every senior node has 500 pre-stored keys. Therefore, the total storage overhead in the scheme is 1000 × 20 + 20 × 500 = 30,000.

**Figure 7:**
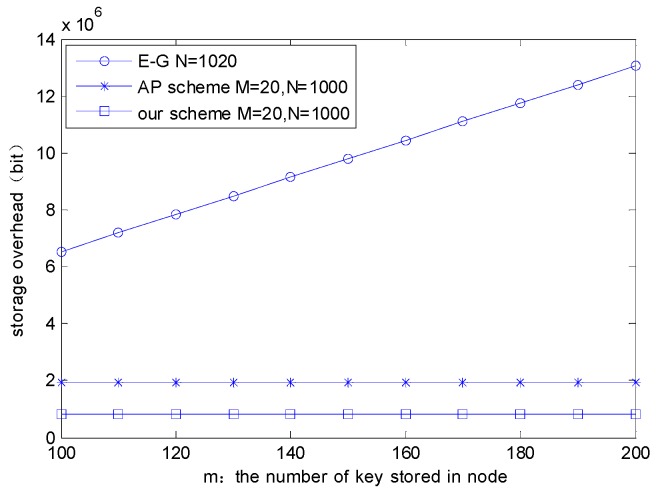
The comparison of node’s storage overhead among E-G, AP, and the proposed scheme after the session key establishment.

Figure 7 shows the comparison of the proposed scheme, E-G scheme and AP scheme on the storage overhead performance after the session key establishment. Although the length of an asymmetric key is longer than that of a symmetric key encryption system, a 160 bit asymmetric key length roughly equals the length of 64 bit symmetric key in security, and the total storage overhead of the proposed scheme is smaller. This is mainly because of the introduction of dynamic clustering and the MS node. In the proposed scheme, communication between clusters is not needed, and cluster heads only need to establish the security of communication with the MS. As a result, each cluster head only needs to store the public key of the node within the cluster and the corresponding session key. It means it does not need to store the public key and corresponding session key for the entire sensor network.

We can conclude from the above that with the same number of keys stored in the nodes, the proposed scheme also has smaller total storage overhead compared with the E-G scheme and the AP scheme as well.

### 4.3. Energy Consumption

**Figure 8:**
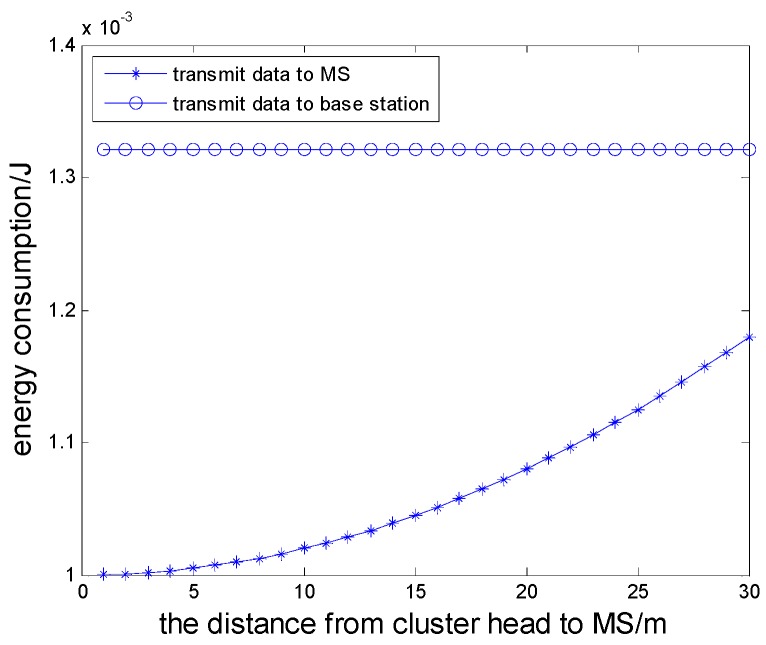
The contrast of energy consumption for transmitting data to the base station and the MS.

**Figure 9:**
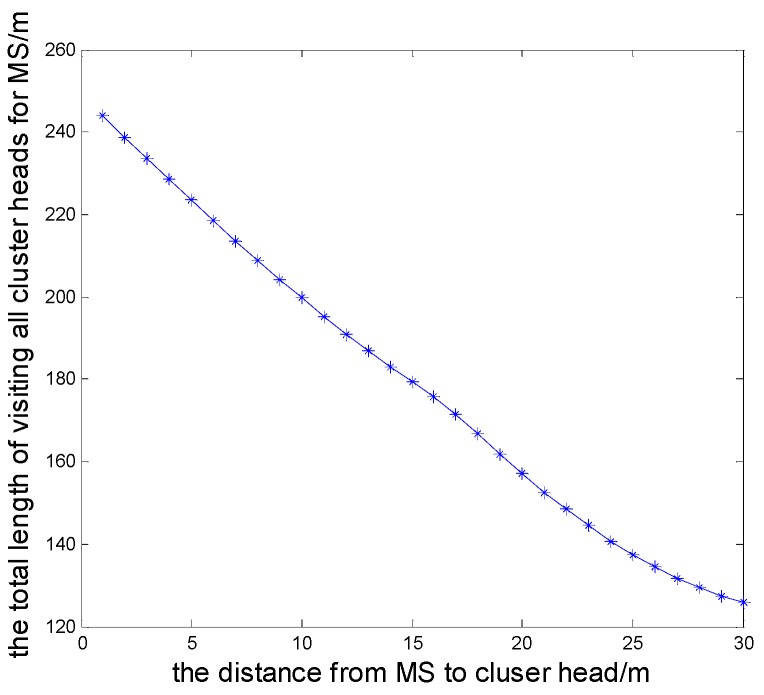
The relationship of the movement distance and stop sites of the MS.

In the proposed scheme, the MS moving model is abstracted to a TSPN model, and that is to say data collection can be undertaken as long as the MS is within the the communication range of the sensor nodes. Compared to the TSP model, this model can reduce the movement distance of the MS and the energy consumption for transmitting data of the cluster heads. Now we let the MS collect data docking at the distance of 1–30 m to cluster heads, and the initial energy is 0.1 J. By computing the energy consumption when the cluster head transmits 4000 bits of data to the MS and a base station, respectively, we can analyze the comparison results and prove that it can effectively save the energy consumption of data transmission when we introduce a MS node. The simulation result is shown as Figure 8. We also give the relationship between the movement distance and the stop sites of the MS, shown in Figure 9. 

In a wireless sensor network, if we do not adopt the MS movement model, the fused information of the cluster head has to be transmitted to thr base station in single hop or multiple hop mode. Data transmission over long distances not only will easily interfere to the data transmission links, but also increase the energy consumption of the nodes. However, the energy consumption of transmission could be obviously reduced after introducing the MS node.

**Figure 10:**
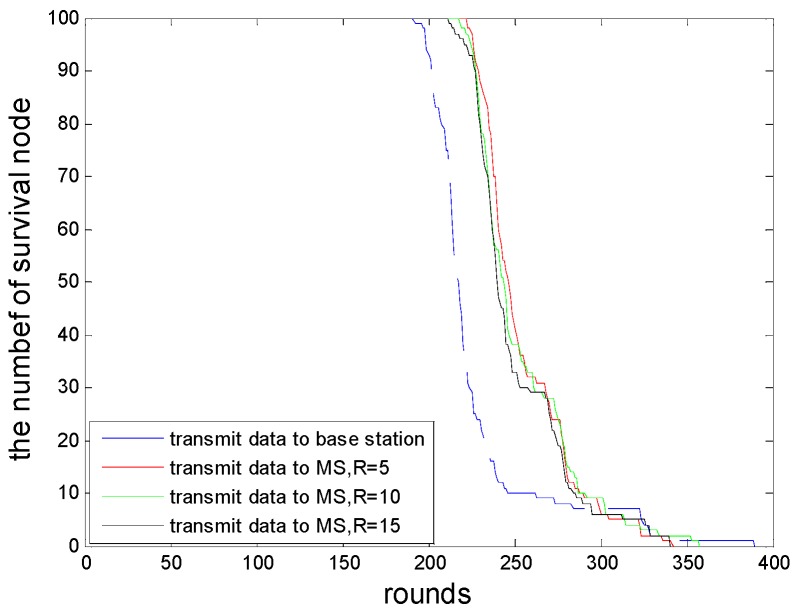
The relationship of stop sites of the MS and the survival time of the network.

In this scheme, the proposed dynamic clustering method makes the network topology change at any time, so the optimal MS route will change relatively. From the point of view of energy analysis, the proposed scheme saves energy consumption. Therefore, it prolongs the survival time of the network. The selection of stop sites of the MS will influence the survival time of the network, and this relationship is shown in Figure 10.

If we define the death time of the first node as the survival time of the network, and the initial energy is 0.1 J, the first node will die in the 222nd, 217th, and 211th round when the MS stops at the distance of 5 m, 10 m and 15 m from the cluster head, respectively, and the first dead node dies in the 191th round when sending the collected data to the base station. As shown in Figure 9, the farther the distance from the stop site to cluster head is, the shorter the movement distance of the MS will be, and if the stop site is far from the cluster head, the round of the first node death will appear ahead of time, and the survival time of the network is shorter. For a MS, the energy of the node is relatively abundant, but from the perspective of security, a node's premature death will easily make the network unstable, and even lead to cracking. Therefore, it is worth extending the survival time of the network at the cost of sacrificing the movement distance of the MS.

### 4.4. Safety Analysis

#### 4.4.1. Data Security

**Figure 11:**
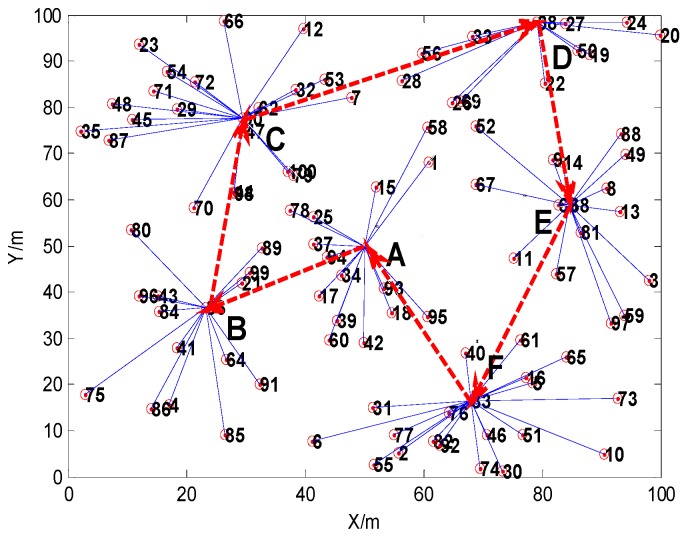
The result of route planning after considering the distance factor.

**Figure 12:**
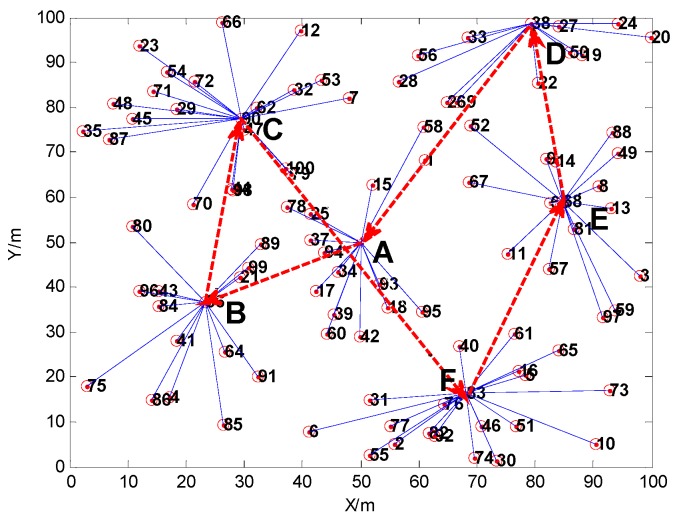
The result of path planning after considering the distance factor and path selection probability.

The result of route planning after considering the distance factor is shown in Figure 11, and the result of route planning based on the Genetic Algorithm considering the relative shortest path and the probability of route selection is shown in Figure 12. In the scenario of time delay tolerance, since the energy is relatively abundant for the MS, the moving distance of the MS may be allowed to increase appropriately. Since the storage space of the cluster head is limited, if the data collected in the cluster head is not taken away by the MS within the prescribed period of time, the data stored in cluster head is likely to overflow or be overwritten. The risk is obvious, especially for the cluster, whose scale is larger, since this kind of cluster head has to buffer more data. Therefore, on the premise of sacrificing movement distance of the MS, the MS should first visit the cluster heads which contain larger amounts of nodes.

For example, from investigating the simulation results in Figure 11 and Figure 12, the total length of the route path in Figure 11 is 249.3480 m, and the sum of the path selection probabilities is 1.1968. Meanwhile, the total length of the path in Figure 12 is 286.2078 m, and the sum of path selection probabilities is 1.2037. The movement distance of the latter has 36.8598 m more than the former, but its sum of the path selection probabilities is greater than in the former. When the MS moves to the cluster head C, the next cluster head will be D if the MS moves follwing the shortest route. However, if we consider the route choice probability factor, the next stop is cluster head F. Because the scale of cluster F is larger compared to cluster D, and the probability of route choice *P_C→F_* is greater than *P_C→D_*, the MS should visit cluster head F preferentially.

#### 4.4.2. Resilience

In the proposed scheme, each sensor node is preloaded a pair of public and private keys before deployment. The session key between the member node and cluster head is established on the basis of the cluster key which is encrypted with the public key of the node in the cluster during the broadcast of the cluster key, and only the corresponding private key of the member node can decrypt and get the cluster key, then they can establish the session keys. The private key of a member node will be used during the process of session key establishment. The method of a pair of scalars on an elliptic curve is adopted in the process of cluster key transmission, and the private keys of the cluster head and MS are also involved in the process. Considering the decryption difficulty for the elliptic curve discrete logarithm and the random large integer *k*, the probability of disclosing the cluster key by calculation will be decreased when the public key *P* and the basis point *G* on the elliptic curve are captured by an adversary. The cluster head and the MS establish a session key with the improved ECDH algorithm, which ensures the forward security of the network, and the forward security means the leakage of any current private key will not affect the keys established before. The session key between the cluster head and MS is established with the random large integer *R*, public key, private key and the basis point *G*, so the session keys established already will not be leaked due to the randomness of the large integer *R*, even if the private key is decrypted.

Any existing key management schemes cannot absolutely assure the security of a sensor network. This article presents a key management scheme based on dynamic clustering and the optimal route choice of the MS, in which the network topology structure and the session keys will be regularly updated. New nodes will be allowed to participate in clustering and establish a session key after their verification by a preset digital signature. The member nodes only need to establish a session key with their cluster heads, and do not need to communicate with any neighbor nodes. This will reduce the influence on them from the link capture of adjacent nodes. In addition, the nodes between clusters will not exchange any information each other, and this could avoid interference from neighboring clusters as well. This can guarantee a limit to the danger that the private key of the nodes in the cluster is leaked within its own link or cluster. In order to impede the goal that an adversary wants to decrypt the session key through long-term monitoring and analyzing data in the network, this scheme adopts the dynamic keys updating policy of “one time one key”. That means the keys update instant in each round of clustering. After the MS completes the traversal for all cluster heads, the base station will launch a new round of clustering, and all keys will be re-established after clustering. This can avoid the use of the same key for data transmission for a long time, and reduce the backward security threat where a pseudo node obtains other nodes’ communication keys and associated information according to the known key’s information:. Therefore, we can summarize the security advantages of the scheme proposed in this article as follows:

1. The analysis of confidential performance: the session keys between the sensor nodes and the cluster heads are established by the cluster key, which is transmitted through a pair of elliptic curve scalars, since cracking the problem of a discrete logarithm on the elliptic curve is a well-known difficult problem, it can ensure the confidentiality of transmissions.
2. The analysis of forward safety performance of the whole network: the session keys between the cluster heads and the MS are established by the random large integer *R*, public key, private key and the basis point *G* all together. Due to the randomness of the large integer *R*, even if the private key is decrypted, the session key established before will not be revealed.
3. The analysis of dynamic security of the whole network: when the MS completes a traversal round, the base station will start a new round of clustering and the topology structure of the network will be also changed as well. At the same time, the session keys between cluster heads and the MS, and the session keys between cluster heads and the member nodes will all be re-established, which can realize the keys’ instantaneous updating in each round of clustering to prevent the pseudo nodes from capturing the information in the data link by long-term detection.
4. The analysis of performance of resistance to possible attacks: the simulation results show that compared to the E-G scheme, AP scheme and q-composite scheme, the proposed scheme has higher connectivity and less storage overhead with the same number of keys stored in each node. When there are external attacks and the malicious nodes try to capture the keys, due to the higher connectivity, any data transmission anomalies are more likely to be detected by the network, and the attacks could thus be identified. In addition, the capture probability of the keys will be lower due to the higher storage efficiency of the keys. On the other hand, the proposed scheme realizes the random dynamic key management by dynamic clustering and optimal route planning of mobile sinks, which can greatly reduce the capture possibility of the keys between the MS and cluster heads and the keys between the cluster heads and the member nodes within the cluster through regular listening by the malicious nodes. Apparently, the proposed scheme could have better resistance to possible attacks, and ensure the network security.

## 5. Conclusions

This paper proposes a key management scheme based on dynamic clustering and the optimal route choice for MSs. The scheme carries on dynamic clustering in the network periodically by the PSO algorithm with multi-objective optimization to change cluster heads regularly. It can avoid the early death of the nodes who forward data chronically, so to some extent it can effectively address the problem of energy holes in WSNs. Also we discussed the problem of MS mobile route planning. The movement distance and the probability of route selection are regarded as the optimized goal. As a tradeoff, after appropriately sacrificing the cost of a certain distance, it can ensure that the cached data in the limited storage space can be read in time, and reduce the danger of overwriting or overflowing ofthe cached data. After fully considering the heterogeneity of resource allocation between the MS and cluster heads, the session key between them could be built by the improved ECDH key agreement method, and the session key between the member nodes and cluster head will be established by a binary symmetric polynomial. The simulation and performance evaluation show that the proposed scheme can provide better resilience to node capture, as well as higher network connectivity and storage efficiency.
